# Ethics of Nudging in the COVID-19 Crisis and the Necessary Return to the Principles of Shared Decision Making: A Critical Review

**DOI:** 10.7759/cureus.57960

**Published:** 2024-04-10

**Authors:** Nancy Junger, Oliver Hirsch

**Affiliations:** 1 Psychology, Independent Researcher, Tübingen, DEU; 2 Psychology, FOM University of Applied Sciences, Siegen, DEU

**Keywords:** shared decision making, nudging, code of ethics, covid-19, conflicts of interest, behavior control

## Abstract

Nudging, a controversial technique for modifying people's behavior in a predictable way, is claimed to preserve freedom of choice while simultaneously influencing it. Nudging had been largely confined to situations such as promoting healthy eating choices but has been employed in the coronavirus disease 2019 (COVID-19) crisis in a shift towards measures that involve significantly less choice, such as shoves and behavioral prods. Shared decision making (SDM), a method for direct involvement and autonomy, is an alternative approach to communicate risk. Predominantly peer-reviewed scientific publications from standard literature databases like PubMed, PsycInfo, and Psyndex were evaluated in a narrative review. The so-called fear nudges, as well as the dissemination of strongly emotionalizing or moralizing messages can lead to intense psycho-physical stress. The use of these nudges by specialized units during the COVID-19 pandemic generated a societal atmosphere of fear that precipitated a deterioration of the mental and physical health of the population. Major recommendations of the German COVID-19 Snapshot Monitoring (COSMO) study, which are based on elements of nudging and coercive measures, do not comply with ethical principles, basic psychological principles, or evidence-based data. SDM was misused in the COVID-19 crisis, which helped to achieve one-sided goals of governments. The emphasis on utilitarian thinking is criticized and the unethical behavior of decision makers is explained by both using the concept of moral disengagement and the maturity level of coping strategies. There should be a return to an open-ended, democratic, and pluralistic scientific debate without using nudges. It is therefore necessary to return to the origins of SDM.

## Introduction and background

Nudging was first described by Sunstein and Thaler in 2009 as an alternative to more restrictive policies such as bids and bans or subsidies, fees, and taxes, as, by definition, it does not restrict freedom of choice [1]. Such policies, which are claimed to preserve freedom of choice based on the practice of nudging would steer people in a certain direction, while still giving them the opportunity to make their own decisions within a given choice architecture. In doing so, nudging has shown to be both cost-effective and potentially helpful in promoting goals, such as public health, in addition to economic goals [2,3]. Examples of nudges in the context of public health measures are as follows: apps that provide feedback on calorie intake, a reminder of a doctor's appointment via a text message, the nutri-score printed on food packages, warning labels on cigarettes, the design of government websites where certain information is highlighted, as well as the "opt-out system" for organ donation (the automatic registration for organ donation from which you have to actively opt out if you do not want to donate your organs). At first glance, this approach appears like an efficient method for solving many problems. Nudging is presented as a gentle aid to decision-making as long as it is used properly. Cass Sunstein emphasizes that "Any official nudging should be transparent and open rather than hidden and covert. Indeed, transparency should be built into the basic practice. [...] In either case, the relevant action should not be hidden in any way. Government decisions in particular should be subject to public scrutiny and review. A principal advantage of nudges, as opposed to mandates and bans, is that they avoid coercion. Even so, they should never take the form of manipulation or trickery. The public should be able to review and scrutinize nudges no less than government actions of any other kind" [4]. However, the use of nudging circumvents the informed consent of the nudgee, thus posing a question on the ethical grounds of this practice in the realm of medicine [5-8]. Other authors argue that the use of nudges instead of informed consent is unethical and should not be implemented [6,9,10]. Doctors nudging their patients might lead to a imbalance of moral considerations, and damage the relationship of trust between the doctor and patient [5]. It is therefore obvious that this is a controversial topic.

Oliver makes the criticism that there is rarely a pure form of nudges and that what are officially called nudges are technically not nudges at all [11]. Shoves are coercive paternalistic measures that are applied where the considered benefits outweigh the perceived costs. A current example of this was the former New Zealand government's plan to ban smoking completely by 2025. Budges are regulatory interventions on the supply side. Instead of selling cigarettes in attractive packages, the manufactures could be forced to sell them in plain packages. Saghai emphasizes that nudges should be easily resistible in order to preserve freedom of choice [12]. There are circumstances that can negatively affect people’s attentiveness and inhibitory capacities. Time pressure, stress, submission to perceived authority, fatigue, anxiety, cognitive load, and distraction are factors that can undermine this easy resistance. Advertising campaigns of the pharmaceutical industry are explicitly mentioned in this context. The term "behavioral prod" is introduced for an influence that uses a default-setting, framing or anchoring to guide people in the desired direction and is therefore not easily resistible and directive [12]. Chriss argues that nudging and social marketing do more harm than good in the long run [13]. A nanny state is emerging that thinks it is better to make decisions for its citizens. A so-called cognitive elite has the assumption that the masses are ignorant and that they do not know what is best for them. Social marketers will not stop at nudges to introduce people to healthier foods. They will expand their techniques to other areas of life like criminal justice, environmentalism, or any other social issue. Chriss cautions that a growing regulatory state will lead to inefficiency, corruption, and to less choice and freedom [13].

In this article, we outline the use of nudges in pandemic management [14], give a detailed account of the suggestions of the German COVID-19 Snapshot Monitoring (COSMO) study and their influence on the pandemic-related policy in Germany and argue that the non-evidence-based use of nudging to manage a global pandemic may have led to unintended yet serious socio-economic, psychological, and health problems, and societal consequences and is thus incompatible with the ethical standards of health and public management. In that regard, it is important to mention that all measures taken by governments to contain COVID-19 are discussed controversially in the literature and their evidence base is more than unclear, and further similar results are still being published [15-27]. As the side effects of using nudges in the pandemic management probably led to serious consequences, which will be described in the following sections, we ask for a debriefing of the nudgee. Otherwise, there is the question of who would take the responsibility of doing harm to the nudgees, and in this situation, possible harm to whole populations. We propose the practice of shared decision making (SDM) as a more ethical alternative to nudging and argue that the core characteristics of the latter [28] are similar to those of propaganda [29] as they are applied in the present form and manner.

## Review

Results

Nudging in the COVID-19 Crisis

So far the use of nudges has been studied primarily in confined everyday life settings, which do not perturb the overall emotional well-being or lifestyle of the nudgee [30]. Previously, the use of nudging has not been a subject of research in a crisis situation of worldwide dimensions, like the ongoing SARS-CoV-2 pandemic that had a substantial impact on the physical, psychological and existential well-being of large portions of the world population [31]. Accordingly, there is no empirical or sound scientific evidence for both its effectiveness and impact in times of particular stress. Some authors even question its general effectiveness [32]. Nonetheless, the practice of nudging has been scientifically recommended and widely adopted by governments across the world [33], for example, to persuade the population to comply with containment measures [34] and get vaccinated [35-37].

As early as March 2020, 681 UK behavioral scientists addressed the UK government in an open letter to express their concerns about the approach to pandemic management, and to offer their support in dealing with the crisis [38]. In it they called for scientific grounding and a rethinking of the government's hesitant strategy to introduce social distancing measures as they had been enforced in the rest of Europe and urged for a radical approach in the form of "high-visibility interventions". Furthermore, the authors stressed the importance of perceived threat and implied that a lack of more drastic measures may undermine the adherence to less strict actions such as hand washing. A parliamentary inquiry subsequently found that the less stringent measures were initially chosen out of concern that people would not comply with tougher measures [39]. As the "scientific consensus" moved toward tough measures such as social distancing and wearing masks in public, nudging was considered a way to get the citizens to change their behavior accordingly even without prior evidence if it would work in an extreme situation such as a pandemic [39]. Nonetheless it was believed that insights from the social and behavioral sciences could help align human behavior with the recommendations of epidemiologists and public health experts [28,33]. For example, van Bavel and colleagues identified several insights for an effective response to the pandemic and emphasized relevant knowledge gaps to which attention should be paid by researchers in the following months. Important topics were related to managing threat, navigating different social and cultural contexts, improving science communication, balancing individual and collective interests, dealing with conspiracy theories, providing strategies for effective leadership, gaining public trust, and offering coping strategies such as social and emotional support [33]. The duration of the containment measures was based on the "transition phase" concept proclaimed by the WHO (until a vaccine or effective treatment was available) [28].

The main principles of the nudging techniques applied in pandemic management are reflected in the MINDSPACE framework put forward by the Behavioural Insights Team (BIT) from the United Kingdom's Cabinet Office [40]. This framework comprises Messenger, Incentives, Norms, Defaults, Salience, Priming, Affect, Commitments, and Ego to promote "prosocial individual behavior".

In retrospect, so-called fear nudges were obviously used in the pandemic situation to reduce the psychological distance to the event [41-44], by manipulation of threat perception, for example, by the use of fear appeals such as "worst-case scenarios" or by using strongly moralizing or emotionalizing messages [33,45]. Fear is said to be an important emotion during a pandemic, which mobilises a set of defensive reactions in the fight against a threat in both humans and animals. Strong fear appeals are associated with the greatest changes in behavior, but only when people have a perceived self-efficacy. Otherwise, low perception of self-efficacy is associated with high levels of defensiveness and leads to helplessness, if people are not able to cope in the threat situation [33]. According to the definition, fear nudges are designed "to evoke feelings of fear, loss and uncertainty", leading the nudgee to engage in an activity supposed to make him feel safe [46]. van Bavel et al. conclude that risk communication should likewise be aimed to deal with the "optimism bias", where people underestimate their risk, without the induction of "excessive feelings of anxiety and dread" [33].

To give an example, according to the first "COSMO" wave of March 3, 2020, the coronavirus was perceived by some people as the "media hype" at the beginning of the crisis, which in turn was associated with a lower perception of risk [47]. The authors described that psychological proximity to the coronavirus appeared to be an important aspect that would influence risk perception and protective behavior. In COSMO wave 9 from April 28, 2020, fear nudging was recommended [48]. The authors explained that the fear of a second wave might be a great motivation for compliance with the current rules. And that this circumstance could be used in crisis communication to encourage compliance with the measures. However, there would remain the risk of a boomerang effect, i.e., if the second wave failed to materialize, there could also be a loss of trust, the authors concluded. The resulting recommendation was that, in well-founded cases, reference should be made to both a possible second wave and associated renewed restrictions. A similar approach was followed in the United Kingdom, where the Scientific Advisory Group for Emergencies (SAGE) gave the advice that "the perceived level of personal threat needed to be increased among those who were complacent by using hard-hitting emotional messaging" [49]. Fear-based communication campaigns are considered effective and are reported to have led to both behavior change and compliance among the majority of the population [33,50]. However, the evidence base of fear appeals is highly controversial, and conclusions about causality cannot usually be drawn due to practical limitations and methodological shortcomings [51]. Similarly, the use of fear appeals neglects potential side effects or impacts on an individual's mental health (e.g., exacerbation of a medical condition), even when the approach has been combined with coping strategies [50-52]. This comes into play especially when the intervention is applied over such a long period of time. These mechanisms can have a negative impact on mental health, for example, by promoting the development of psychological distress such as anxiety, guilt, and shame [53-61]. Nevertheless, the use of fear appeals or fear nudges in public health or policy persists and is even promoted [51]. Table 1 shows that people have suffered from the containment measures and that mental health has deteriorated significantly over the course of the pandemic. There is reason to suspect that fear nudging and/or the manipulation of threat level played a significant role in this, which should urgently be the subject of future investigations.

**Table 1:** Studies showing negative effects of the pandemic crisis management on mental health

Study design	Results
Systematic review [62]	The prevalence of psychological distress was 13.29%; there was a high prevalence of depression (16%), anxiety (15%), insomnia (24%) and PTSD (22%).
Nationwide population-based study [63]	Anxiety in children doubled from 15% (pre-pandemic) to 24%-30%.
German nationwide COPSY study of mental health and quality of life in children and adolescents [64]	A total of 71% of children and adolescents felt burdened by the contact restrictions; 65% experienced school and learning as more exhausting; more escalating arguments between children and parents (37%) were seen; there was a decline in the health-related quality of life (15% reported low quality of life pre-pandemic vs. 40% in the pandemic); the risk of mental health problems rose from about 18% to 30%.

We describe the potential direct and indirect relationships between fear nudges, containment measures and negative health effects in more detail below based on the assumption of a biopsychosocial model [65,66]. Fear nudges led to more psycho-physical stress, and thus more psychological and somatic harm. They influenced people to stay socially isolated by repeatedly showing shocking images of intubated patients in intensive care units implying this as a direct consequence of social interactions, e.g., children meeting their grandparents. Social isolation and the feeling of loneliness have negative biological effects as they activate the same brain structures as the feelings of craving and hunger [67]. Isolation is a great health risk factor and could be a predictor of mortality [68-72]. In relation to COVID-19, the risk of dying with COVID was even higher when anxiety was high, which can also be a result of fear nudges [73]. Anxiety and fear-related disorders were the second highest risk factor for death among the underlying conditions considered in this study. Mental health problems not only lead to a potential life loss of 10 years [74], but also have the consequence of more deaths by suicide [75-79]. Walker et al. estimated that 14.3% of deaths worldwide (which would be eight million deaths each year) were attributable to mental disorders, counted before the COVID-19 pandemic [74]. During the pandemic, the rate of suicidal ideations became much higher and may result in higher suicide rates in the future [80].

As the intention of fear nudges was a higher compliance with pandemic policies, like staying at home, avoiding going outside, and maintaining social distance to public facilities, people were more likely to stay longer periods at home, often in small rooms with their families. This led to a higher conflict and aggression potential and to the higher use of domestic violence against children and women. These side effects resulted in a high prevalence of harm [81-85].

Since fear nudges are designed to induce feelings of fear, they lead to a higher experience of stress by activating the stress system. This mechanism plays a role in the etiopathogenesis of several diseases [86]. Stress affects not only the mind, but also the body by activating in many complex ways the immune and nervous system and has therefore somatic consequences. There exists a connection between mental stress, (neuro-)inflammation and negative somatic effects, for example, with the role of cytokines and hormones [87]. Already in 1956, Selye examined the pathways of the hypothalamic‐pituitary‐adrenocortical axis (HPA) and the sympathetic‐adrenal‐medullary (SAM) system in the pathomechanism of stress. He described exhaustion after experiencing chronic stress as dangerous and potentially hazardous [88]. The danger of chronic activation of the stress hormone system results in an increase in overall diseases, such as adrenal insufficiency [89], cancer [90], major depressive disorder (MDD), diabetes, and metabolic syndrome [91,92]. The metabolic syndrome is defined by the WHO as a pathologic condition consisting of abdominal obesity, insulin resistance, hypertension, and hyperlipidemia. This syndrome has become the major health hazard in the modern world, increasing morbidities like type 2 diabetes, coronary diseases, and stroke. Saklayen states that the total cost of the condition including the cost of health care and loss of potential economic activity is in trillions [93]. Fear nudges not only increased psychological stress and therefore triggered an activation of the HPA and the SAM system with the described increased morbidity, but also led to worse health behavior with less sports/exercising, higher consumption of unhealthy foods like sweets, and more time on digital media, thereby increasing obesity and consequently elevating risk factors for metabolic syndrome [64].

As there exists a bi-directional network between the brain and the cardiovascular, immune, endocrine and other systems, there are several effects of mental stress on both psychological and somatic health functions, e.g., a smaller hippocampus in relation to stress-related conditions and mild cognitive impairment in this context [94]. There is actually evidence that the telomeres, a form of stress-response biomarkers, shorten with exposure to psychosocial stress, which leads to more somatic and psychiatric illnesses and a probable shortening of life [95]. Some researchers have already coined the term "COVID-19 anxiety syndrome" [53,61]. As depression has risen by imposed countermeasures during the COVID-19 pandemic and fear nudges are suspected of having supported this, it should be known that increased depression is linked to a worse immune system situation and more harm and somatic illnesses [96,97].

The overall increase in the perceived threat level in order to persuade the population to comply with the regulations probably has contributed to persistent fear during the COVID-19 pandemic. This has an impact on our future society as this fear did worsen the health status of young parents [98-100] and of children and their development [101]. The experience of distress, especially in young children, can lead to pathological effects on brain structures and thus to a higher prevalence of mental and somatic disorders and symptoms [102-107]. A higher stress experience in childhood is connected to higher morbidity and mortality inducing a loss of life expectation [107].

Next, we would like to exemplify the use of affect to influence the public compliance with the imposed policies to contain the SARS-CoV-2 virus. This was done by means of moral-emotionalizing slogans such as "Stay home. Save lives." [108,109], "Don't kill your grandma" [110] or "Don’t let a coffee cost a life", etc. [111]. Messages with moral-emotionalizing content are perceived more effectively [40] and have the potential to be spread more quickly through social networks, a phenomenon called "moral contagion" [33,112]. According to the MINDSPACE "Messenger" principle, health organizations and health professionals used Instagram and Twitter to share public health messages [40]. The Centers for Disease Control and Prevention (CDC) has created a social media toolkit with sample messages and accompanying graphics and videos bearing the CDC logo for Twitter, Facebook, Instagram, Pinterest, and LinkedIn [40]. The introduction of social norms such as "protect yourself and others", "do not endanger others", or appeals to "solidarity", which are usually consistent with people's moral values [14,33,35,113,114], are other examples of moral nudges, as well as the idea that vaccination is a social contract [115], and one has a moral obligation to be vaccinated for the common good. Those who do not abide by this social contract run the risk of being socially sanctioned or ostracized [115,116] while those who comply with it are socially rewarded. The underlying assumption seems to be that the willingness for cooperation is more persistent, if non-cooperative behavior can be punished [117]. The MINDSPACE principle "Norms" used observational learning via social media for the creation of new norms in the own social group (e.g., by watching others in the own social network, avoiding social gatherings, or engaging in hand hygiene) [40]. To summarize, discrimination, scapegoating [118] and conformity pressure [119] appear to have become acceptable means of bringing about behavioral change. To conclude, the implementation of a "new normal" [28,43,120] that is constantly around, equipped with signs full of rules and ground markings, social distancing notices [121], mask requirements [116,122,123], tests [124,125], and contract tracing apps [126], in principle, contains elements of "permanent nudging" to achieve the goal of a "large-scale behavior change" [43].

Another example is the use of aerial drones, helium balloons with cameras, "home quarantine" stamps on travellers’ forearms and disinfection tunnels, or a so-called nationality nudge, a speech by the head of state, which prompted the people of India to go into lockdown [120]. All taken together, one can no longer speak of preserved freedom of choice in the context of the pandemic, since the measures are ultimately linked to sanctions as recommended, for example, by the COSMO study of the University of Erfurt, Wave 4, March 24, 2020: "Social norms should be communicated and violations of the rules sanctioned; it is important to know that others also abide by the rules" [127]. In Texas, violation of mask mandate was penalized up to $1000. In other states, violations of stay-at-home orders have been punishable by fines ranging from $100 to $25,000, and in some regions, up to 18 months in jail [40]. Some experts felt that "nudges" were not enough and called for more coercive interventions such as vaccination passports and different isolation measures for the unvaccinated and vaccinated in order to encourage people to get vaccinated, which were abstractly referred to as "pushes and shoves" [39]. Following the MINDSPACE principle "Incentives", people were rewarded for maintaining social isolation by "carrots" (as named by the authors), for example, governments subsidizing impactful home-based work, the provision of free online courses, or access to web-streamed entertainment services [40]. Taken together, this represents a reference to behaviorist punishment and reward scenarios that should have led to an open-ended discussion in a pluralistic democratic society.

COSMO: Pro-government Nudging?

There are a large number of "Nudge Units" worldwide; for example, they are in Canada, New Zealand, Ireland, and Germany, built on the concept of the Behavioural Insights Team (BIT) in Great Britain [128]. Governments around the world have more and more implemented behavioral insights in their policy making, and receive professional support from behavioral insights experts from international institutions such as the Organisation for Economic Co-operation and Development (OECD) [129]. The WHO's newly implemented Behavioral Insights Unit published the so-called "WHO tool for behavioural insights on COVID-19", which maps, among other things, the effect of risk communication on the population at regular intervals [130,131]. This tool is a low-cost, easy-to-use instrument for monitoring, among other things, knowledge, risk perception, behavior, acceptance of measures, stigmas, and public trust in institutions and government [41]. It is also intended to be flexible in handling dynamic situations. Based on regular surveys of the population, recommendations can be flexibly adjusted. At the time of writing, 13 countries and regions were participating in the WHO tool for behavioural insights on COVID-19, also known as "COSMO, or COVID-19 Snapshot Monitoring" [132]. Since 2020, the WHO has a "Technical Advisory Group on Behavioural Insights and Sciences for Health". Professor Susan Michie, Health Psychologist and Director of the "Centre for Behaviour Change" (CBC) at University College London (UCL) [133], was appointed chair of this group in July 2022 as successor to Cass Sunstein. Her "Behaviour Change Wheel" (BCW) [134] seems to be one of the theoretical foundations of the Scientific Advisory Group for Emergencies' (SAGE’s) COVID-19 behavior change interventions [49], and it was likewise one theoretical basis for some items in the COSMO survey tool [130]. The BCW framework is based on "Sources of behaviour" (opportunity [social, physical], motivation [automatic, reflective], capability [psychological, physical]) in a green-colored inner circle, followed by a red-framed "Intervention functions" circle (restrictions, education, persuasion, incentivization, coercion, training, enablement, modeling, environmental restructuring), and the outer grey-colored “Policy categories” circle (guidelines, environmental/social planning, communication/marketing, legislation, service provision, regulation, fiscal measures).

In Germany, the COSMO group of the University of Erfurt and the Bernhard Nocht Institute for Tropical Medicine [135], respectively, is scientifically responsible for behavioral insights monitoring and has been providing recommendations in the context of risk communication on the COVID-19 pandemic since March 2020 based on regular cross-sectional surveys of the population [14,49,136]. The COSMO recommendations thus have a direct impact on politics and the media, for example, via the Science Media Center Germany [137], as well as, due to the expertise in the field of health communication, via statements of the Leopoldina [138], and recommendations of the Expert Council of the Federal Government [139]. Therefore, the role of the Science Media Center and the Leopoldina in risk communication and crisis management during the pandemic should also be examined more closely.

First, it seems appropriate to take a closer look at the COSMO study conducted in Germany from a methodological perspective. Participants were recruited through an online service that conducts paid surveys [140]. People without Internet affinity and with language barriers were almost completely excluded; people with special financial needs and thus in special life situations were automatically overrepresented. The latter is highly problematic psychologically: if one is dependent on compensation from the survey, the pressure to answer in a socially desirable manner may be more easily increased than if one is completely free of it. Respondi further says that only those who are honest and whose opinions are meaningful are included in the Access Panel and that they constantly check identity, plausibility and response behavior that suggests the suspicion of unknown data cleanups. The study authors state that it is a repeated cross-sectional monitoring and the participants in each survey change. Nevertheless, direct comparisons are made between different measurement time points, and measurement points in graphs at different measurement time points are connected with lines. This is methodologically incorrect. The different measurement points are not comparable as this is not a longitudinal study with an identical sample. It is claimed that the participants correspond to the distribution of age, gender (crossed), and state (uncrossed) in the German population. However, the age of the participants stops at 74 years, so representativeness cannot be assumed. The actual most vulnerable group of the oldest population is thus not reached at all by the survey. In addition, the question arises to what extent the best interests of the child (mental health and basic child needs) were fundamentally taken into account since the COSMO study is based on an adult sample (see also, "Limitations of the study") [130]. The following recommendations and interpretations criticize parents who are critical of the measures and would thus make their children uncomfortable. School closures are recommended although children were not consulted. Thus, it is spoken about instead of speaking with children and adolescents. Beyond this, the serious and partially far-reaching negative effects of recommended school closures and other restrictive measures on children and adolescents were not sufficiently taken into account [141]. Other problems include the use of leading questions, display problems on cell phones, and the purely exploratory regression methods used to analyze the data.

Different recommendations of the German COSMO study at different points in time will be analyzed exemplarily with regard to ethical, psychological, and evidence-based criteria (see the Appendices). The original documents can be read in detail [42,135].

In summary, major recommendations of the German COSMO study do not comply with ethical principles, basic psychological principles, or evidence-based data, and should therefore be rejected. The collected data should be reviewed by an independent panel of scientists and scientifically appropriate conclusions and recommendations should be derived.

Shared Decision Making: A Forgotten/Misused Paradigm?

The foregoing has shown that a principle such as nudging, which from its origin was supposed to allow freedom of choice, was used during the COVID-19 crisis to restrict precisely that freedom of choice in order to get the population to comply with measures set by governments. The side effects, like worsening of health conditions through fear nudges, e.g., with experiencing more loneliness, anxiety, depression, increase in domestic violence, eating more unhealthy food, exercising less and therefore being at a higher risk of physical morbidity by developing metabolic syndrome, were not communicated to the population.

This must be judged ethically questionable and, strictly speaking, no longer qualifies as nudging in the original sense. In doctor-patient communication, shared decision making should be anchored as a concept to allow patients more autonomy and self-determination and also offer information regarding harms and potential side-effects. Since the COVID-19 crisis involves medical decisions, SDM should be the standard procedure and we critically review whether this standard has been maintained.

In shared decision making, patients are empowered in a way that they actively ask questions and participate in decisions about their health care on the basis of their preferences and values. In clinical practice, patients are not regularly informed about criteria of SDM, e.g., being presented several options [142]. Sepucha and Mulley mention evidence that poor communication, lack of knowledge, and neglect of patients' preferences are often found in the context of medical decision making [143]. Even patients' understanding of the provided information is rarely checked. The authors emphasize that a treatment decision cannot be solely based on clinical guidelines; patients' values and preferences are also important. The model of a rational decision maker does not appear appropriate for medical decisions as emotions play an important role and depend on the importance such decisions have for the individual patient. Table 2 displays results from different publications dealing with SDM concepts; it can be demonstrated that a unified concept of SDM still does not exist.

**Table 2:** Main results of publications dealing with SDM concepts SDM, shared decision making

Author/study design	Main results
Review by Makoul and Clayman [144]	A total of 31 different concepts of SDM were identified. Most authors regarded SDM as a process. There is debate about whether the resulting decision should be equally shared, or if sharing the process is the goal. An integrative model was proposed in which the discussion is either initiated by the physician or the patient. Sharing a decision should be represented on a continuum. Essential elements are as follows: definition and/or explanation of the problem, presentation of options, discussion of pros and cons, inclusion of patient values and preferences, discussion of patient ability and self-efficacy, discussion of physician recommendations, checking of understanding, making or deferring decision, and arrangement of follow-up.
Model by Elwyn et al. [145]	A simplified model for SDM in clinical practice was proposed, with three steps: choice talk (patients are made aware that reasonable options exist), option talk (more detailed information about options is provided), and decision talk (patients explore their preferences and connect them to their decision).
Medical decision making [146]	A distinction between classic paternalism, passive informed consent, active informed consent, SDM, and normative SDM is made with the help of decision aids.
Evidence-based medicine and SDM [147]	Sharing information is seen as an important part of SDM. Evidence-based medicine is an essential prerequisite for SDM.
Treatment decision aid [148]	Communication of uncertainty is an essential element of SDM as the outcome of therapeutic options is generally probabilistic. Patients with higher decisional uncertainty are reported to be more involved in decision making.
Review on SDM key components [149]	A consensus on what SDM is has still not been reached. Describing treatment options with unbiased, neutral information, making decisions, caring for patient preferences, tailoring information, deliberating, creating choice awareness, and learning about the patient were key components in the reviewed studies.

Scheibler et al. presented results where about half of the patients desired to be involved in decision making, but a much smaller amount was actually involved [150]. The willingness of physicians to involve their patients in decision making is considerably lower than the preference of their patients. Attitudes towards SDM are generally positive in providers and patients, but this does not consequently translate into the implementation in clinical practice [151]. Positive aspects of tests and treatments were more likely to be discussed than negative ones that, however, could represent a bias. Sharing evidence and considering patient preferences were important aspects in the SDM process. Those patients with greater knowledge and higher involvement in SDM reported a higher matching with their goals and concerns. They also felt more positively about the outcome of their treatment [151]. Patient-related characteristics did not show a clear association with the occurrence of SDM, including such obvious parameters like age or education [152]. More implicit psychological parameters might influence the occurrence of SDM, and unless these are specified, one has to conclude that SDM can be performed with any patient. Another review found that trusting physicians increased the probability of engaging in SDM although unconditional trust should be regarded as negative as a collaborative relationship has to be established in SDM [153]. Also, patient characteristics like better physical health, higher self-efficacy, higher decision-making skills, younger age, and higher health literacy were associated with higher participation in SDM. A decisive factor was physicians' communication skills. Ineffective SDM was characterized by a lack of humanistic concern. Organizational support, like the financial compensation of additional time, is needed for physicians to implement SDM [153]. Physicians were less supportive of SDM in situations where they thought that well-evidenced guidelines favored one treatment over another [154]. On the other hand, SDM was supported in situations where treatment options carried significant risks. In some of the reviewed studies, it was the concern of the physicians that the involvement of the patients could lead to "wrong" or "irrational" decisions. Pollard et al. mention that SDM is often vaguely defined and not properly understood. Physicians still think that SDM is a process in which they should convince or try to persuade the patients, or they regard it as a simple information transfer. Patients' lifestyle, abilities, values, and goals have to be incorporated in an SDM process [154].

Since SDM can be viewed as an open-ended process, it therefore does not seem compelling to consider the level of patient outcomes. This was done by Shay and Lafata with regard to affective-cognitive, behavioral, and physiological patient outcomes [155]. When patients reported that they participated in SDM, they were more likely to show better affective-cognitive outcomes. Behavioral and health outcomes were less likely to be positively affected. The authors nevertheless emphasize the importance of SDM that encompasses an "…ethical mandate to protect patient autonomy and self-determination, an interpersonal benefit of promoting trust in the patient-clinician relationship, and an educational gain of increasing patient knowledge about treatment options, benefits, and harms through a SDM process" [155]. However, SDM, comparable to the discussion of the original concept of nudging, is also increasingly being used to achieve "desired" outcomes, which then, however, no longer constitutes SDM. Sanftenberg et al. did a systematic review and meta-analysis on the impact of SDM on influenza vaccination rates and explicitly stated that SDM processes might be an effective strategy to increase influenza vaccination rates [156]. Apart from the fact that there were high heterogeneities concerning the use of SDM and the actual vaccine uptake in the included studies, which shed doubt on the results, this certainly does not represent SDM in its original meaning as an open-ended process. It becomes particularly irritating when SDM is mentioned in the context of increasing COVID-19 vaccination rates [157]. The authors state that the aim is to present information in a neutral way, so it should also be mentioned that COVID-19 is a disease that primarily affects vulnerable people, especially the elderly. Given the side-effects of the vaccines [25], the risk-benefit ratio appears to be not acceptable especially for younger age groups who predominantly have a mild course and a low infection fatality rate [158]. Instead, the goal is to reduce "vaccine hesitancy" and the authors even talk about SDM being helpful in the context of motivational interviewing if COVID-19 vaccination may one day be mandatory. The authors' recommendation is diametrically opposed to the core of SDM as coercive measures undermine any patient autonomy. The authors also presented results of a meta-analysis that, according to their conclusions, showed that SDM is a helpful and ethically defensible strategy to increase vaccination rates [159]. They stated that they define SDM in a way that "the concerns, personal circumstances, and contexts of patients and their families play a major role in decisions", which emphasizes the autonomy of the patient and the open-ended nature of SDM. However, as they proceeded, they completely changed this reasoning and tried to work towards a preconceived goal, which is not SDM. They say that one should respond to and manage issues regarding vaccine hesitancy and that SDM interventions can be used to address vaccine hesitancy. The authors then refer to the uptake of COVID-19 vaccines and even mention the serious side effects of blood clots, which might even increase vaccine hesitancy if patients were not given information weighing these risks against the risk of contracting the virus. If SDM delivers evidence-based, trustworthy information, then people should be informed that COVID-19 is mainly a disease that predominantly affects older people and/or is a serious disease for those with major complications and that the vaccines may have serious adverse effects [23-25,160-163]. Therefore, the aim of SDM should not be to promote a mass vaccination campaign in COVID-19 as this would be unethical in the light of the massive risk differences in different age and morbidity groups [158,164].

The development of a decision aid regarding Comirnaty vaccination in France went in a similar direction. The authors based their decision aid just on the regulatory study that was published in the *New England Journal of Medicine* [165] and which was criticized for several methodological problems [16,23,166]. They already stated as a goal formulation that SDM could help to reduce vaccine hesitancy that violates the principle of SDM as an open-ended process. They further wanted to incorporate the results of an Israeli observational study that had to be criticized because of massive protocol violations as healthcare workers and nursing home residents were subsequently excluded from the original protocol [167]. It is not stated by those who developed the Comirnaty decision aid if they also presented the absolute risk reduction for a symptomatic COVID-19 infection that was just around 1% in COVID-19 vaccines and was therefore an important piece of information in the decision process [168]. SDM in context with vaccination has clear similarities with persuasive communication. The latter attempts to trigger desired behavior in the other person on a voluntary basis. However, it is clear that the persuader is trying to change the behavior in a direction he or she desires. Therefore, the persuader works with a concealment of purpose [29], which is not compatible with the ethics of SDM.

Early in the COVID-19 crisis, however, there were other positions that advocated for equal patient involvement. It soon became apparent that mechanical ventilation in intensive care units led to a higher mortality in COVID-19 and that realistic information on alternative treatments like conservative oxygenation strategies could have been much more beneficial and could have been the basis for patients participating in their own healthcare decisions [169]. In spring 2020, the Federal Council of Medicine in Brazil considered the prescription of drugs like hydroxychloroquine possible in the context of a shared decision-making process [170]. Westphal and Ramos further argue that SDM respects the autonomy of patients and ensures care consistent with their values and preferences, especially when there are uncertainties and risks associated with an intervention. Physicians should not present options as recommendations as the relationship has to be egalitarian, empathetic, and respectful. Formulating direct recommendations would mean to impose the values of physicians on decisions that have to conform to the lives of the patients. It may also be the result of the SDM process that the decision is postponed [170]. Direct personal involvement of family members in SDM processes in neurological intensive care patients is also emphasized. To this end, the restricted visiting policies were considered an obstacle with negative consequences on brain-injured patients [171]. In a non-representative, mainly female and well-educated sample in Germany, participants were asked about their preference of SDM regarding COVID-19 [172]. In contrast to other findings, older people and those with physical health conditions expressed a higher preference of being involved in COVID-19 decisions. The authors also presented a triage vignette and assessed if participants would be willing to accept that healthcare professionals would be the exclusive decision makers in this situation. They justified this saying that triage has frequently become a reality in the pandemic by erroneously citing just a perspective article published in March 2020 in the *New England Journal of Medicine* with conjectures instead of real data [172]. This has to be interpreted in the context of fear induction as there was never even one case of triage in the German healthcare system during the COVID-19 crisis in view of the non-existent overload of the German healthcare system [173].

In their commentary, Wald and Kelly state some interesting facts on medical consent [79]. The British High Court has ruled that if the consent procedure is started too close to a surgery, this invalidates the consent altogether. The General Medical Council (GMC) guidance on consent emphasizes that patients need to understand benefits, risks, and alternatives of medical procedures and that a dialogue that results in a shared decision between patient and doctor should be encouraged. The mere signing of a form does not ensure that these standards were met. Multiple options are more rarely presented than single recommendations as physicians think that they give up some of their authority. This is critical because for most healthcare decisions, there is more than one obvious option [174]. Kunneman et al. criticize that clinicians engage in technical and formal forms of SDM instead of enacting genuine conversations in which real human interactions take place [175]. Developing an understanding of the person and their situation and establishing ways that make sense for each individual in a specific situation should be the goal of SDM. This should be achieved by humanistic communication paying respect to the dignity of each person, interacting with respect, humanity, compassion, integrity, and empathy. Just about 9% of studies included in their review mentioned aspects of humanistic communication within SDM. The studies primarily focused on the proper application of SDM techniques, but without empathy, SDM techniques might be executed formally in a correct manner but turn out to be ineffective. In a clinical interaction, SDM is too often used to satisfy external requirements like signing a consent form instead of incorporating aspects such as kindness and understanding [175]. Educational programs to teach SDM to medical trainees had only small effects on knowledge, attitudes, and comfort with SDM. High levels of clinical expertise in clinical communication together with an approval of the underlying ethical principles are needed to achieve SDM skills. This is complicated by different definitions of SDM [44]. In their Cochrane review to determine if interventions targeting patients, healthcare professionals, or both patients and healthcare professionals increase the use of SDM, Légaré et al. found low to very low evidence [176].

Discussion

Ethical Aspects

In light of the above-mentioned views, the following questions become unavoidable: who actually decides what is the "right" thing to do? And do adult people need to be nudged and/or "educated" paternalistically? Can adults not be trusted to act on their own responsibility? In the evaluation of the legal bases and measures of pandemic policy by an expert committee to the German government, it was stated, "Participation also involves taking criticism and skepticism seriously and actively dealing with them. Dissenting opinions were often hastily condemned during the Corona pandemic. Those who suggested alternative solutions and approaches were often sidelined without sufficient discourse. In the long term, it will be difficult to successfully manage the pandemic without openly dealing with differences of opinion. If controversy in a crisis situation is not seen as an obstacle but as an opportunity, this contributes to constructive, respectful debate, which is essential in a democratic system" [177]. The measures taken by the German government were criticized as being based on insufficient data and on unclear scientific evidence. There was no evaluation of risk communication with the public that was instead presented in a top-down way. Therefore, the measures presented by governments as having no alternative, that did indeed have alternatives suggested, turned out to be non-evidence-based and must be classified as inaccurate or misleading in their message character, such as the statement by U.S. President Joe Biden that one can no longer be infected with COVID-19 if one gets vaccinated [178]. Against this background, the question also arises whether a detailed risk-benefit analysis of potential (short-, medium-, and long-term) negative effects due to the interventions was made before implementing the measures or applying behavioral insight techniques [179-181]. Mandated measures that interfere so massively with natural human behavior and experience [182] and that have negative consequences, such as social distancing [68,83,180,183,184], mandatory masking [18,185,186], lockdowns [187-189], contact restrictions [67,190,191], access restrictions, school closures [180,192-194], etc., should be reviewed in terms of their hazard potential before they are used and in the course of their application.

Following the MINDSPACE principle "Affect", inducing empathy for those most vulnerable to the virus would encourage self-isolation and social distancing [40]. To summarize, nudges based on fear and moralization can have a negative impact on mental health by promoting the development of psychological distress such as anxiety, guilt, and shame [53-61]. On one hand, people were repeatedly made to believe that they posed a potential life-threatening danger to others, and on the other hand people needed interpersonal contact and closeness, which is part of human nature and existentially needed for people's wellbeing [182]. Additionally, people were under constant stress due to pandemic control measures, fear of the virus, and fear of sanctions. Taken together, this can sometimes result in massive internal conflicts and thus promote the development or worsening of mental illnesses. Mental illnesses, in turn, are statistically associated with reduced life expectancy [74]. Governmentally mandated policies and measures that have the potential to negatively affect mental health, among others, must be subjected to critical scrutiny. There is no question that it is fundamentally unethical to deliberately frighten children and adolescents or to teach them that they are a danger to others, especially their loved ones. In this way, they are exposed to an enormous psychological burden and moral overload, which can be accompanied by massive feelings of guilt. The consequences for their further development are unpredictable [195]. Pandemic control measures are aimed at long-term behavior change on the basis of behavioral insight techniques [196], but people have not been explicitly educated about the use of nudging, about the duration of its application, and about possible negative side effects [10]. This can be compared to the trolley dilemma based on utilitarian thinking. In the most famous variant, the trolley is about to crash into a group of five people who will die as a result of the collision. However, there is still a switch between the trolley and the people. Switching would direct the trolley to another track where there is only one person. It is discussed if it is morally justifiable or even imperative to operate the lever of the switch [197], which would be forbidden in Germany according to the constitution, because in that case, the person is objectified as an obstacle to rescue the other persons, and that violates the his/her dignity as well as one's right to live.

According to Schulze Heuling, there seems to be a "blind spot in the debate" concerning the potential deadly consequences of COVID-19 policies [197]. Thus, both the virus and the measures can have harmful consequences for health, in the worst case even death, which is a dilemma situation that leads to the question, whether it is morally legitimate or even necessary to sacrifice human lives to save others [197]. As an example of instrumental harm caused by the containment measures, the author cites the correlation between the lack of exercise and its implications for health due to lockdowns and social isolation, as well as the known consequences of unemployment and recession, the worldwide increase of poverty, and psychosocial consequences, to name just a few. The so-called Aviation Security Act of 2006 from the Federal Republic of Germany prohibits the shooting down of aircraft misused as weapons on the grounds of violation of human dignity and the right to live (see "Luftsicherheitsgesetz", 2006) [198]. Schulze Heuling explains that the deontological ethical approach forbids the killing of others, even in situations where more lives could be saved than lost. She concludes with the argument that "saving human lives is not a justification for measures that foreseeably result in the death of people" [197], which would make the imperative "to protect life" or "protect others" absurd. Nevertheless, in the course of pandemic management, utilitarian decisions were made worldwide, for the "greater good", and thus so-called instrumental harm was accepted. Even if the intention behind these consequentialist decisions is to save as many people as possible, which assumes that the well-being of each person is equally important, or in other words, so-called impartial beneficience [199], it still remains damaging to the human being. But not intervening would also be immoral, according to Schulze Heuling, which is why she proposes the "focused protection" approach [197]. This approach known as the "Great Barrington Declaration" is mainly about the targeted protection of vulnerable people [200,201]. In proposing the focused protection approach, Schulze Heuling points out that the right to self-determination and the human dignity of the individuals concerned must nevertheless be respected. Therefore, it seems important to give preference to voluntary interventions such as focused protection [202]. It is striking that victims of pandemic response measures, or so-called collateral damage, received little attention and empathy [203]. Those who expressed divergent, empirically based points of view were met with radical intolerance and authoritarianism [203]. However, this raises the question of who determines what ultimately constitutes "truth". The so-called "fact checkers", most of whom do not have any epidemiological training, have very often spread exactly the opposite of facts.

With regard to the COSMO study under consideration, the question arises, to what extent the authors attempt to scientifically substantiate their recommendations. Coercive measures against the unvaccinated were apparently intended to be underpinned by a model calculation that involved the study authors, which accused the unvaccinated of having mainly caused the current COVID-19 crisis [204]. These claims were shown to be scientifically unfounded [205,206]. The complex modeling assumes unrealistic vaccine effectiveness, and claims an unrealistic faster recovery of vaccinated individuals after infection, which represents vaccine failure for them [204]. It was further argued that vaccine status identification (VSI) is associated with societal polarization and that mandatory regulations to get vaccinated could help to increase social cohesion as vaccination status will then become a less important part of one's self-concept [207]. This is a paradoxical and dystopian argument, since the authors assume that compulsory measures can be used to increase social cohesion, although a numerically not insignificant group of society is then to be forced to undergo a medical intervention that they consciously do not want, but this group is then forced to accept this and is prepared to simply give up their vaccination status as the intentionally unvaccinated. It has to be further mentioned that there are completely contradictory positions concerning mandatory vaccinations [208]. The proponents of mandatory vaccination argue that non-vaccination is a sufficient condition of a disaster and that by mandating mass vaccination, the disaster can be prevented. It is further argued that the overriding of individual freedoms could not result in a disaster of a different kind. All of these conditions can be regarded as false. Mandatory vaccination is unethical because it violates body-autonomy and discriminates against innate, healthy biological characteristics. As vaccination is an irreversible medical procedure and not just a behavioral preference, it is not comparable to mandatory seatbelts. This constitutes a trivialization of a medical procedure [208]. Moreover, being given the choice of either getting vaccinated or losing your job instead is not a free decision in the sense of voluntary informed consent, which is the bioethical basis for any medical intervention [10]. This kind of nudging strategy can, from our point of view, be understood as a form of (informal) coercion [209,210]. Informal coercion refers, for example, to the use of manipulation, threats, negotiations, persuasion, pressure, deception or incentives [210,211]. The term is used in psychiatric treatment to distinguish it from "formal coercion" such as forced medication [212]. Even at the end of the pandemic, the authors of the COSMO study do not include in their considerations that COVID-19 is a disease with lower infection fatality rate than previously thought [158,213], and they still demand higher vaccination coverage of the population, even in children and adolescents, contrary to evidence-based findings [214]. They claim that "misinformation" is being spread about the vaccines, while at the same time they are assigning inflated efficacies compared to the actual empirical results [16,23,215]. The recommendations should also be made against the background of the massive conflicts of interest and the equally massive misconduct of the pharmaceutical industry in the past [216]. Moreover, the frequently mentioned late effects in the form of "post-COVID" are the subject of controversial empirical debate [217,218]. Before further engaging with the media, as suggested by the authors, to encourage the population to increase their willingness to vaccinate, there should be a critical analysis of the campaign in public.

We assume that crisis management is based on good intentions; people should be protected from the severe effects of a virus. Nevertheless, the question arises as to how these harmful measures could have come about, despite the duty of care. Conceivable factors and mechanisms that might have contributed to this imbalanced crisis management, which has also caused a great deal of damage on an individual and social level, will be discussed further.

Maturity Level of COVID-19-Related Coping Strategies

As described above, the measures taken in the pandemic to shape and direct opinion have had profound effects on the health of the individual and society (e.g., increase in mental illnesses). We can also see characteristics and effects in the use of risk communication and nudging tools that we might otherwise observe in immature, pathological levels of psychological functioning, e.g., in the use of splitting and black-and-white thinking, or the use of idealization and devaluation, which are known as immature defense mechanisms in psychoanalysis. Since the use of immature, insufficiently thought-out or dysfunctional coping strategies in crises like the pandemic causes more illness and damage in the long term than mature, integrating and constructive solution finding, it is important to ask what kind of coping was chosen in pandemic management. If coping is based on an immature, poorly developed structural level, the basis for finding solutions is already pathologically designed. Coping strategies show cognitive processes and actions that have the goal of overcoming problems, e.g., stress. Depending on the person and the situation, different coping strategies can be chosen.

In the transactional stress model according to Lazarus, a distinction is made between problem-oriented and emotion-oriented coping [219]. Problem-focused coping describes an action with the focus on counteracting stressors and solving them. The stressor is thus the primary target of these coping strategies, which would correspond to a mature personality and a developed society, or as one would expect from mature coping. However, emotion-focused coping is used primarily to calm and regulate the own emotional state. Thus, the primary aim here is to improve the emotional state triggered by the crisis and stress. An attempt is made to reduce unpleasant emotions and feelings of stress or to replace them with other emotions. It is then no longer a question of finding a factual, neutral solution to the problem. Therefore, no long-term healthy solution to the situation can be achieved by using emotion-focused coping. For example, the use of nudges can serve the idealized perception of oneself as "right", "solidary" and "helping" and thus suppress unpleasant feelings such as fear, helplessness, guilt or anger. The unpleasant feelings are then projected, according to psychoanalytical models, onto people who decide or act differently. This leads to the devaluation of those who think differently, onto whom guilt, anger, etc. are shifted [220]. The use of healthy, functional or dysfunctional, pathological coping strategies determines the maturity and development of an entire society here. Therefore, the form and character of the communication, influence and handling of the problems taken, as well as the measures chosen, have a considerable impact on the development of maturity and health of the societies that implemented them, as well as on their following generations.

What are the characteristics of a mature, constructive, healthy way of dealing with crises? According to Kernberg and the object relations theory, the central task in the psychological development towards maturity is the integration of opposing emotions and their connections with self and object representations into increasingly complex units [221]. The differentiation and simultaneous endurance of different points of view as well as the attempt to find compromises and work out an integration of opposing impulses can be understood as a mature way of dealing with crises, the only stable way of coping in the long term. This means that paying attention to and perceiving different points of view and the inclusion of different aspects is of decisive importance in order to reach a constructive solution for the benefit of all. This is opposed to splitting, i.e., idealizing an idea while devaluing, suppressing, despising or punishing opposing aspects, which are characteristics of immature solution strategies that lead to more pathologies in the long run [222]. In contrast, with the use of nudges like "Stay home. Save lives." or "Don't kill your grandma", one may suppose the use of splitting as it indicates that persons who leave their homes or visit their grandparents would be murderers and the others life savers. This ignores at the same time the aspect of basic human needs, humanity and life-enforcing pro-social skills and behaviors. It ignores the fact that isolation of a grandmother during long periods of quarantine is also life-shortening [222,223]. In this sense, these nudges may indicate splitting, or, from a milder perspective, at least they do not show a mature coping that would try to integrate these complex, disturbing and ambivalent aspects.

Splitting is shown in polarized views of self and others and arises due to intolerable conflicting emotions. A person or a collective employing splitting may idealize one aspect (seeing this part as "all good") and devalue another (seeing it as "all bad") at the same time [222]. Primitive denial may have occurred with the fact that important scientific results and studies that showed a contrary conclusion or aspect as the one promoted by international governments were ignored or devalued, which leads to the primitive defense mechanism of primitive devaluation by blaming critical scientists or naming them with phrases as "covidiot" or "conspiracy theorist". Projective identification is often experienced by the person being projected onto as a subtle pressure to behave or believe in a particular way [224]. In the dimension of the social pressure on taking the COVID-19 vaccines to be in the "good part" of the society, or nudging people by interpreting the vaccine as the only way to get "back to normal" and to be rewarded as a "responsible adult", forcing them by using prohibitions and other pressures on the non-vaccinated, it is easy to see how one may feel guilty or similar to a murderer with their decision against the vaccine. Nonetheless, this sensation of guilt is not rational in this sense, as we see in scientific results regarding effectiveness of the vaccines [225]. It is an irrational feeling that could be interpreted as the primitive defense mechanism of projection and projective identification.

In her descriptive study about media headlines in the first months when COVID-19 was declared as a pandemic, Ebrahim states that in international COVID-19 pandemic coverage, consequence frames were used that dramatized loss of life instead of deploying frames of reassurance that foregrounded the high survival rate of this disease [226]. Also, language was modified and changed, e.g., with the neologisms "covidiot" and "vaxxie" (selfie taken while being vaccinated) [227]. New metaphors and terms of war became normal. The "war" of governments, people, and whole societies against the coronavirus was waged with public health "weapons" such as distancing, isolation, or the wearing of medical masks [226]. One-sided arguments and viewpoints were promoted, among others, by using soft power techniques such as nudging, while scientific results of critical aspects were ignored or devalued. This status of black-and-white thinking, idealizing one aspect and invalidating the other, can be interpreted as functions of immature coping. Invalidating people who made their decision against the COVID-19 vaccine as "all the rest" and "enemies of the state" [228] is part of primitive defensive mechanisms [229].

All these examples may indicate severe, pathological psychological mechanisms. The effects on whole populations of such a prospect are immense and must be reflected in order to achieve the best developments and decisions.

Moral Disengagement

One of the concepts that can be applied to explain the imbalances in crisis management is that of moral disengagement by Bandura [230]. This concept tries to explain why people behave inhumanely and still feel good about themselves. This is possible because they really believe that their harmful actions are effective to prevent suffering, so they disengage morality in their execution [230]. The mechanisms of moral disengagement are moral justification, euphemistic labeling, advantageous comparison, displacement of responsibility, diffusion of responsibility, disregarding, distorting, denying harmful effects, and dehumanization. Even harmful means are implemented with moral justification as decision makers are indeed convinced that they are morally superior to their critics. Euphemistic language is used to cover up the harmful effects of their own measures. Those negatively affected by the non-evidence-based interventions against COVID-19 were called "collateral damage" and received little or no empathy [203]; the restructured environment, from which it was impossible to escape, was described as the "new normal"; and face masks were called "everyday masks", just to name the most prominent examples. Advantageous comparisons were made in the sense that it is said that the measures, which turned out to be non-evidence-based when viewed from an empirical viewpoint, prevented more suffering than they caused [230]. Displacement and diffusion of responsibility emerged in terms of subordinates claiming that they were just carrying out orders to impose draconian measures and a social atmosphere of hatred and agitation could develop against people who, for example, exercised their democratic right to decide against COVID-19 vaccination, and so they were even threatened with financial disadvantages and imprisonment. The disregard, distortion, and denial of harmful effects can be seen by the fact that the governments constantly denied negative effects of their measures, e.g., lockdowns [27], and currently they are tolerating negative side effects of the COVID-19 vaccines [16,231]. Dehumanization has been an essential ingredient in making possible a social exclusion of those who claim their legitimate right to refuse vaccination against COVID-19. These were labeled, for example, "freeriders", "all the rest”, "social parasites", "racist", and not being part of society [228]. As we know from world history, it is easier to act brutally against people when they are not viewed as human beings.

Utilitarianism as Moral Justification

The underlying moral approach in crisis management is basically a utilitarian one, which has been emphasized in particular in relevant scientific papers on crisis management [28,33]. Habersaat et al. described that public health approaches are "often utilitarian in essence", meaning that they are intended to maximize the overall benefit to the population [28]. The chosen moral concept could support Bandura's theory of moral disengagement in relation to these undesirable and worrisome developments in crisis management by serving as a moral justification that psychologically exonerates those responsible. This is because, following utilitarianism, instrumental harm, which means "the sacrifice of an individual to save a greater number" [232], is tolerated in order to protect public health [28]. According to experts in the field of moral decision making, utilitarianism has a neglected positive altruistic core [232]. The willingness to act for the good of society differs culturally and may be more pronounced in collectivist than in individualist countries, where the focus is on achieving advantage for the individual [28]. According to the authors, these cultural differences influence attitudes towards measures and make it difficult to predict their acceptance in different areas of application. Balancing utilitarian values intended to benefit the common good with individual rights such as equality and personal dignity can also raise difficult issues, for example, involuntary quarantine. Taken together, public health measures should respect fundamental rights, such as "freedom of speech, privacy, due process of law, freedom from discrimination and freedom of religion" [28]. Measures that are perceived as unjustified can undermine both public support for crisis management and general confidence in the authorities. Backlash against harmful misinformation related to freedom of speech can vary country-wise. However, in general, they conclude that the continued adjustment of the response strategy should be maximally respectful of rights and human dignity. In contrast, the COVID-19 pandemic management was based on social engineering, paternalism, behaviorism and collectivism [233], and supported strategies that restricted fundamental human rights and violated what is actually inviolable human dignity. According to Schulze Heuling, complex dilemma decisions concerning life and death irrespective of the ethical approach lead to massive psychological stress and excessive demands, and it is not surprising that the people responsible both tend to act against their better judgment and try to justify their actions [197].

In conclusion, a predominantly one-sided view has come to bear, which has reduced people mainly to their behavior. This is reminiscent of a classic behaviorist view of human beings, which assumes that the inner human experience represents a kind of "black box" or is based on a "stimulus-response model".

Other Conceivable Explanatory Approaches or Factors

Other possible explanations such as the false consensus bias could also be partly responsible for the worrying developments [234]. The false consensus bias is the excessive overuse of self-referential-related knowledge in estimating the prevalence of attributes in a population. The bias gives the impression of being statistically correct, but instead merely mimics normative inductive reasoning. Similarly, the human tendency to act, although it is predictable that an action is useless or even harmful, is called "action bias" [235]. Psychological stress and excessive demands with high responsibility, and panic reaction, might be another possible explanation. Also, imaginable would be that lack of empathy, diffusion of responsibility, too great a power imbalance due to emergency laws, scientism, and conflicts of interest could have led to this imbalance. The point is that it was known that the majority of the measures taken to ensure compliance through nudging would entail high social, psychological, and economic costs [28]. In November 2020, the WHO stated that decisions to strengthen, alleviate, or implement pandemic measures "must be weighed on the impact that such measures have on societies and individuals". For instance, they considered impacts on economy, security, mental health, psychosocial well-being, human rights, food security, treatment of diseases, socioeconomic inequalities, healthcare programs, and public sentiments [236]. Authors should discuss the results and how they can be interpreted from the perspective of previous studies and that of the working hypotheses. The findings and their implications should be discussed in the broadest context possible. Future research directions may also be highlighted. Societal polarization [237, 238] has to be avoided and de-escalation should be prioritized [239]. This can be achieved with adequate communication [240] and not in paternalistic messages [241] that might even induce opposite effects [242].

## Conclusions

It has become apparent that the massive censorship of opposing scientific viewpoints at all levels of the information society has ultimately led to societal polarization. It was shown that vaccinated people display discriminatory attitudes towards unvaccinated people similar to those expressed towards immigrants and minorities. It should therefore be the task of state authorities to be active in the de-escalation of conflict between different social conflict groups. For future situations of this kind, it is imperative to learn from this to return to an open-ended, pluralistic scientific debate instead of claiming to represent "the science" and using nudges, behavioral prods, and shoves to get the population to only behave in the way desired. It is therefore necessary to return to the origins of shared decision making and implement at least basic elements such as choice talk, option talk, and decision talk with elements of balanced, open risk communication that respects patients' values and dignity, and gives them the right to reject offered treatments without disadvantages at any time. However, barriers to implementing SDM could include time constraints, less empathetic practitioners, and people who want the decision to be taken away from them. Although there is still no agreement on the key components of SDM, describing treatment options with unbiased, neutral information, caring for patient preferences, tailoring information, deliberating, creating choice awareness, and learning about the patient should become the standard practice. Interdisciplinary and mature coping strategies should be the base of any crisis management. Both sides of the dilemma should be taken seriously (negative impact of a threat vs. negative impact of counter-measures) avoiding weighing up life against life. Regular risk-benefit analyses and evaluation of the measures should also be implemented for this purpose.

Even if, in addition to questionable fear nudges or threat manipulation and emotionalizing messages, beneficial health behavior such as physical activity was nudged as part of crisis management (which was not discussed in detail in this review), a discussion must take place about whether this paternalistic approach in general is appropriate for public health crisis or any other events of disaster. The trend to take over decisions for people in a paternalistic way in a pandemic situation induces the concept of learned helplessness as an escape or avoidance and is regarded as a depression-like coping deficit in averse but avoidable situations. Promoting a society in which the individual gives up responsibility towards a power holder results in a trend toward less democracy and more collectivism. One may argue that the actions taken during the pandemic may not accurately represent true nudging strategies but rather a distortion or misapplication of the concept. As such, it may be essential to recognize that the negative assessment of "nudging" during the pandemic may not be applicable to nudging as a whole. The deceptive labeling of these strategies as nudging may rather have been a part of the framing campaign and played a significant role in shaping public perception and response. However, we believe that in addition to these mislabeled instances of "nudging", there were also instances of basic nudging strategies being employed, unbeknownst to the public. This raises significant ethical concerns, particularly in such exceptional circumstances. A comprehensive multidisciplinary review, investigation and reappraisal of crisis management, risk communication and the role of the media during the corona crisis, as well as clarification of motivation and conflicts of interest of persons capable of and responsible for making decisions during this time are essential to avoid similar fundamental mistakes in the future. To avoid further unforeseeable consequences from the use of nudging or behavioral insights in emergencies, and to ensure greatest possible transparency, we also emphasize the urgent need for discussing both the regulation and the introduction of accountability for the use of nudging techniques in general, especially in the field of public health.

## Appendices

**Table 3:** Evaluation of exemplary statements and recommendations of the COVID-19 Snapshot Monitoring (COSMO) study by the University of Erfurt

Date (wave)	Statements/recommendations	Evaluation
March 24, 2020 (4th wave)	Violations of the rules should be sanctioned. It is important to know that others also abide by the rules.	Self-responsible action is apparently ruled out from the start.
March 31, 2020 (5th wave)	Large parts of the population are willing to wear masks in public. It must be strongly pointed out that masks cannot prevent infection.	At that time, the authors of the study must also have been aware of the correct non-evidence base for wearing masks in public.
April 14, 2020 (7th wave)	Transparent communication is important to maintain trust.	This is the exact opposite of what was done in the German crisis management (see, for example, the application of fear nudges).
April 28, 2020 (9th wave)	Cloth masks convey a limited protection.	Apparent intention to prepare for a mask obligation with masks from other manufacturers.
May 5, 2020 (10th wave)	The maintaining of new routines should be supported and behavioral scientists can provide valuable tips here.	This means the use of nudges with the support of behavioral scientists, which should then result in a so-called new normal.
May 12, 2020 (11th wave)	Transparent risk communication on expected benefits and risks of vaccination against COVID-19 is important and risk communication should be managed.	Risks of the vaccine were not conveyed by official bodies to the public. Other experts in their field who wanted to implement a real transparent risk communication were scapegoated and defamed [238].
May 19, 2020 (12th wave)	New, necessary behaviors should become routines and habits. Behavioral support measures and findings from the behavioral sciences should be used for this purpose.	Measures that have not been reviewed for evidence and have not previously been recommended by the WHO in a pandemic situation have been identified as necessary, and are to be applied using behavioral science techniques without explicit consent of the population. This is to be seen as an enormous ethical problem.
May 25, 2020 (13th wave)	Cities or counties where there were more than 50 new infections per 100,000 population should significantly limit public life in the region.	The number of 50 per 100,000 was never a number based on study evidence and was thus arbitrary.
June 9, 2020 (14th wave)	The number was even reduced to 35 so-called infections per 100,000 population.	This number was also non-evidence based. From the beginning, there could be no mention of infections, since it was primarily a matter of subjects with a positive SARS-CoV-2 PCR test result who were essentially symptom-free and therefore mostly without clinical significance [243]. To propose drastic restrictions in the sense of lockdowns also lacked any evidence [27].
July 7, 2020 (16th wave)	There was implementation of infographics in shops and public transport.	There was further intrusion into the spheres of citizens' lives and spreading fear.
September 1, 2020 (20th wave)	Conspiracy theories should not just be countered on the content level. This is not specified in more detail.	Not just some of the statements made by critics labeled as conspiracy theories were true in the further course. The authors list the thesis that the SARS-CoV-2 virus is man-made as a conspiracy theory even though the U.S. Department of Energy recently concluded as such [244] and clear indications have emerged that this virus could be a released pathogen from a laboratory accident [245,246].
September 30, 2020, and October 1, 2020 (22nd wave titled "Special evaluation pandemic fatigue")	In general, evidence-based information on viral and bacterial load under masks should be provided, and recommendations for action to avoid increased viral and bacterial load under masks should also be provided, if appropriate.	Mask-wearing in public has no real evidence basis, also because people do not follow the most elementary hygiene rules in the context of mask-wearing, resulting in massive self-contamination [247]. A necessary daily change of masks to even avoid self-contamination is completely unrealistic for a large part of the population.
October 13-14, 2020 (23rd wave)	Emotional aspects are also important, as they activate behavior; however, fear appeals aimed at triggering fear should be discouraged, as these are more likely to lead to counter-reactions.	This is in sharp contrast to politics and media coverage, which from the beginning have spread fear and panic among the population and defamed corrective, reassuring information as trivialization [248]. Furthermore, it contradicts the COSMO recommendation of wave 9 from April 28, 2020, in which it was recommended to use the concern of the people about both a possible next wave and associated restrictions in risk-communication as "motivation" to comply with the measures [48], which we understand as a fear nudge or fear appeal.
October 27-28, 2020 (24th wave)	Asymptomatic infection is mentioned. Communicative measures on the relationship between an early significant caseload reduction and the expected length and effectiveness of a lockdown are claimed to be reasonable.	The evidence base for asymptomatic infection is highly questionable [249]. The claims regarding lockdowns have also since been refuted by empirical data [27,250].
November 10-11, 2020 (26th wave)	Using the 3G rule (tested, convalesced, vaccinated) could avoid becoming part of a superspreader event.	There is no sound empirical evidence for this claim either [249,250].
December 8-10, 2020 (29th wave/"First focus survey on vaccinations and lockdown")	Leopoldina is currently calling for measures to combat the pandemic to be drastically tightened; 60% agree that there should be a hard lockdown as soon as possible to significantly reduce the number of cases; 29% reject this, and the rest are undecided. A total of 23% think the current measures are excessive (previous week, 27%). Approval of the restriction of civil liberties has risen from 40% (previous week) to 53%. We also know from the previous week's survey(s) that the introduction of regulations (e.g., compulsory masks, lockdown) has led to a rapid and significant change in behavior.	It should be pointed out here that the scientific director of the COSMO study was also a member of the Leopoldina working group that advocated for stricter measures and hard lockdown during the winter holidays of 2020/2021. These harder measures were recommended as the current measures were thought to be not sufficient to achieve the reduction of new infections, while at the same time incurring high social and economic costs and psychological stress [251]. This is only one example that makes the approach of weighing up different goods against each other during the pandemic to solve a moral dilemma very clear. A partial lockdown is to be replaced by a hard lockdown, which will also have serious consequences [252-254]. And since these hard measures were not effective as described above, they did not minimize collateral damage, either.
December 15-16, 2020 (30th wave)	Not only the benefits but also the limited reliability of rapid tests should be communicated more widely.	Methodological problems of rapid point-of-care tests, especially regarding false positives, have also been shown [255].
End of December 2020 (waves 31 and 32)	Knowledge that rapid contact reduction is relevant to caseload reduction should be spread.	This is in contrast to findings that there are no associations between the strength of measures and the outcome [177].
October 15-27, 2020, and January 4-12, 2021 (waves 1 and 2 of COSMO Thuringia)	Citizens should be actively supported in seeking information on the risks and benefits of COVID-19 vaccination during the period leading up to the vaccination offer to enable the best possible evidence-based decision and to debunk any misinformation that may arise.	One-sided information was officially always presented that portrayed this experimental medical procedure as highly effective and risk-free [256], although there was little or no evidence for these claims [16,24,25,257,258]. A critical debate did not take place. Vaccination was presented as the only way out. Alternative drug treatments were defamed from the beginning.
February 16-19, 2021 (36th wave titled "Second vaccination focus survey")	Information from the Paul Ehrlich Institute (PEI) is cited that claims that AstraZeneca's COVID-19 vaccine is highly effective and would prevent COVID-19 disease in the majority of cases. It is further stated that vaccine reactions occur relatively frequently after the administration of the vaccine. They are supposed to be short-lived and usually reflect the body's normal immune response to vaccination. According to the PEI, each individual vaccinated person benefits from the expected protective effect [259].	This has to be considered as incorrect, even as disinformation. AstraZeneca's vaccine was withdrawn from the German market at the end of 2021 (see [16,24,25,257,258]).
February 23-24, 2021 (37th wave)	Education about the importance of effectiveness and that all vaccines provide equally high, near-perfect protection against severe courses and death seems important.	This can also be regarded as incorrect (see, for example, [16,24,25,257,258]).
March 9-10, 2021 (38th wave)	Importance of regular rapid testing and self-testing for pandemic control. Government communication alone no longer reaches a sufficiently large group due to the loss of trust and other actors should urgently be involved.	The mass testing of healthy, asymptomatic people is highly controversial epidemiologically and in no way leads to pandemic control [255].
March 23-24, 2021 (39th wave)	Achieving acceptance of the measures can hardly be a political goal at present.	This conveys the character of an underlying autocratic attitude, since one is apparently not willing to sufficiently justify measures to citizens through consistent evaluation and to negotiate with them.
April 6-7, 2021 (40th wave)	An understandable communication of vaccination risks is proposed.	This is in contrast to the German Ministry of Health that denied any risks from the vaccinations at that point in time.
April 20-21, 2021 (41st wave)	Participatory approaches should also be pursued more strongly, as approximately 40% of respondents would like to participate in decisions.	Instead of responding to this, the government continued to pursue a one-sided paternalistic top-down communication that regarded citizens as immature.
May 4-5, 2021 (42nd wave)	Vaccination of children and adolescents can contribute to herd protection. Extraneous protection by vaccination is still assumed.	It is now clear that there can be no herd immunity from this vaccination and that children and adolescents do not benefit from this vaccination due to minimal to mild courses of COVID-19 [158]. Even at that time extraneous protection was clearly refuted by mass vaccination failure [260].
May 18-19, 2021 (43rd wave)	The authors are obviously aware of the lack of effectiveness of these vaccinations, as they point to contradictions in public communication that arise due to the discrepancy between the hoped-for and the actual lack of effectiveness of vaccines: "I get vaccinated to hug my grandchildren again" vs. "Vaccinated people have to keep their distance". Mandatory vaccination cannot increase vaccination readiness and may lead to psychological side effects, such as skipping other voluntary vaccinations.	There is no herd immunity from these vaccines and vaccine failures were apparent from the beginning. Therefore, mandatory vaccination should have never been a point of discussion.
June 15-16, 2021 (45th wave)	It should be communicated more clearly how vaccinated people contribute to pandemic control and how vaccination can enable the path to normalcy.	According to current data, this can be labeled as incorrect information. It is morally questionable to put people thirsting for normality under this type of pressure and this can be considered a form of informal coercion [209,261], because it contradicts informed consent of the free will.
June 29-30, 2021 (46th wave)	Since confidence in the safety of the vaccination was the most important factor for the willingness to vaccinate, the greatest possible care should be taken in communication (e.g., regarding a possible connection with the 2nd BioNTech vaccination and an increased incidence of myocarditis, or when it is communicated that a more extensive recommendation is not given "because data on safety are lacking", this can be understood by parents as "is dangerous").	In the further course of the vaccination campaign, increasingly severe side effects occurred [16]. Manipulative, persuasive communication should be considered here, as emerging safety signals may be downplayed by such statements in the absence of long-term studies in an experimental medical intervention.
July 13-14, 2021 (47th wave)	The claim that vaccination with both BioNTech's and AstraZeneca's vaccines almost certainly protects against hospitalization should be communicated more forcefully. That vaccination reduces transmission, even if it does not prevent it completely, should be more clearly communicated. The authors further state that presenting incidence by vaccinated and unvaccinated may increase the perceived effectiveness of vaccination.	The separate presentation of incidence by vaccinated and unvaccinated was discontinued again due to data scandals that led to complete miscalculation of incidence among the unvaccinated [262]. This was because people with positive SARS-CoV-2 PCR tests with unclear vaccination status were simply included with the unvaccinated. Furthermore, the highly questionable definition of "vaccinated" should be criticized, which is 2 weeks after full "immunization", so that categorization as "unvaccinated" occurs before that. All these data manipulations can be seen as further nudging and propaganda techniques to push vaccination in the population.
November 2-3, 2021 (waves 54 and 55)	An explanation of measures is seen as still being important (e.g., that 2G [vaccinated or convalesced] is primarily an emergency infection control measure because then the virus spreads less, not a nudge to the unvaccinated). Compulsory education should be considered. COVID risks would still be underestimated by the unvaccinated; there would also be a lack of clarity or knowledge about the social benefits. There is a need to confirm non-vaccination (so-called default regulation): vaccination is the rule, and those who do not want it must have it signed off by a physician after an educational discussion and present it where defined necessary.	Untested vaccinated subjects pass on the virus just as unvaccinated subjects do [263], and their privileging under 2G was at no time justified from an epidemiological point of view. COVID-19 is primarily a disease that affects older people, and/or a serious disease for those with major complications and the majority of the population is at low risk [158,164]. Therefore, the recommendation regarding compulsory education is not comprehensible. There is no social benefit of vaccination since no external protection in the form of sterile immunity is achieved [215]. The coercive default regulation measure (or "default nudge") is incomprehensible from an evidence-based perspective and thus appears ethically questionable.
November 30 and December 1, 2021 (waves 56 and 57)	Convalescents should be educated early about the purpose of vaccination and any fears should be allayed through active education. Mandatory vaccination should be discussed and planned as a measure if other measures fail to achieve sufficient vaccination.	Studies attest better protection to convalescents than to vaccinated persons [264], so an additional vaccination recommendation of convalescents is not indicated. Those who execute their democratic right not to take an experimental medical procedure are labeled from official bodies as "vaccination refusers" and it is still assumed that vaccination is the only way to end the current situation [265]. Neither the indication for mass vaccination nor other treatment options are discussed in any way. One insists on an imaginary herd immunity, which must be achieved with an unspecified high vaccination rate. This must be rejected as a non-evidence-based approach.
December 14-15, 2021 (58th wave)	The likely increasing (absolute) number of serious cases should be communicated in the context of the Omicron variant. It is called the "Omicron variant of concern".	Information from South Africa, where this variant was first observed, already showed at the beginning that it is a harmless variant with low dangerousness [213].
December 15-27, 2021 (COSMO panel wave 1)	Considerations of compulsory vaccination enforcement will have to take into account that among the unvaccinated, there is a large proportion of individuals who wish to avoid the obligation. The trust of the unvaccinated in the federal government is already so low that it can hardly be eroded.	It remains unclear, however, what form these considerations should take, as they can ultimately only result in further coercive and control measures. In a democratic and pluralistic state, this should actually give pause for thought, and instead of stigmatization and a policy of exclusion, one's own measures should be evaluated for evidence and checked to see whether such coercion is ethically justifiable at all [118].
February 22-23, 2022 (60th wave)	Extraneous protection from vaccination continues to be assumed. The handling of physicians who advise against vaccination should be discussed within professional societies.	There was mass occurrence of COVID-19 vaccine failure that was already present at that time [266].
Up to wave 70, dated November 29-30, 2022	Vaccination should continue to be made as easy as possible. Confidence in the safety of vaccination has decreased.	A very offensive vaccination strategy continues to be recommended even after the failure of mandatory vaccination in the German parliament and contrary to empirical data. Reference is made only to the authors' own "evidence-based" resources for improving vaccination communication, without including new peer-reviewed results that fundamentally question the current vaccination strategy.
